# Researches on the Erectile Organs of the Female

**Published:** 1859-04

**Authors:** Charles Rouget


### RESEARCHES ON THE ERECTILE ORGANS OF THE FEMALE.

BY M. CHARLES ROUGET.

Translated by THOMAS BEVAN, M.D., Chicago.

M. CHARLES ROUGET has lately published a somewhat remarkable monograph, entitled, "*Researches on the Erectile Organs of the Female and the Tubo-ovarian Muscular Apparatus, in their relations with Ovulation and Menstruation;* the results of which researches are as follows:

1. That in the female the uterus presents the structure of an erectile organ, a veritable corpus spongiosum.

2. That to the ovary is also annexed an erectile bulb.

3. That in all classes of the vertebata, and in particular in all the mammalia, a special muscular apparatus embraces the oviduct and the ovary, and determines their adaptation.

4. That the fascia of the ovario-tubar muscular membranes (*mesoarium* and *mesometrium*) have with the spongy bodies, and especially with their different sinuses, such relations, that at the moment of contraction the meshes of the network, in the midst of which the venous conduits make their way, contract in all directions, by which these last are necessarily compressed, and the exit of the blood more or less completely prevented.

5. That the contraction of the ovario-tubar muscular apparatus, continuing during the whole period of ovulation, the obstacle to the exit of the blood, and the erection of the spongy body of the uterus and the ovary which results, have the same duration.

6. That menstruation also coinciding, on the other hand, with ovulation, it is natural to consider it as the immediate consequence of the erection of the uterus. A real menstrual hemorrhage does not exist elsewhere but in such organs as present a true erectile structure.

7. That if the sexual excitation can, as it appears probable it may, determine the erection of the uterus and ovary, it is easy by this to account for its influence in bringing together the periods of menstruation and ovulation.—*Journal de la Physiologie.*

